# Phytochemical constituents, nutritional values, phenolics, flavonols, flavonoids, antioxidant and cytotoxicity studies on *Phaleria macrocarpa* (Scheff.) Boerl fruits

**DOI:** 10.1186/1472-6882-14-152

**Published:** 2014-05-08

**Authors:** Ma Ma Lay, Saiful Anuar Karsani, Sadegh Mohajer, Sri Nurestri Abd Malek

**Affiliations:** 1 Institute of Biological Sciences, Faculty of Science, University of Malaya, 50603 Kuala Lumpur, Malaysia; 2 Univerisity of Malaya Centre for Proteomics Research (UMCPR), University of Malaya, 50603 Kuala Lumpur, Malaysia

**Keywords:** Cytoxicity, Phytochemicals, Nutritional values, Flavonoids, Flavonol, Phenol, Antioxidants, *Phaleria macrocarpa* (Scheff.) Boerl fruits or *P. macrocarpa*

## Abstract

**Background:**

The edible fruits of *Phaleria macrocarpa* (Scheff.) Boerl are widely used in traditional medicine in Indonesia. It is used to treat a variety of medical conditions such as - cancer, diabetes mellitus, allergies, liver and heart diseases, kidney failure, blood diseases, high blood pressure, stroke, various skin diseases, itching, aches, and flu. Therefore, it is of great interest to determine the biochemical and cytotoxic properties of the fruit extracts.

**Methods:**

The methanol, hexane, chloroform, ethyl acetate, and water extracts of *P. macrocarpa* fruits were examined for phytochemicals, physicochemicals, flavonols, flavonoids and phenol content. Its nutritional value (A.O.A.C method), antioxidant properties (DPPH assay) and cytotoxicity (MTT cell proliferation assay) were also determined.

**Results:**

A preliminary phyotochemical screening of the different crude extracts from the fruits of *P. macrocarpa* showed the presence secondary metabolites such as of flavonoids, phenols, saponin glycosides and tannins. The ethyl acetate and methanol extracts displayed high antioxidant acitivity (IC_50_ value of 8.15±0.02 ug/mL) in the DPPH assay comparable to that of the standard gallic acid (IC_50_ value of 10.8±0.02 ug/mL). Evaluation of cytotoxic activity showed that the crude methanol extract possessed excellent anti-proliferative activity against SKOV-3 (IC_50_ 7.75±2.56 μg/mL) after 72 hours of treatment whilst the hexane and ethyl acetate extracts displayed good cytotoxic effect against both SKOV-3 and MDA-MB231 cell lines. The chloroform extract however, showed selective inhibitory activity in the breast cancer cell line MDA-MB231 (IC_50_ 7.80±1.57 μg/mL) after 48 hours of treatment. There was no cytotoxic effect observed in the Ca Ski cell line and the two normal cell lines (MRC-5 and WRL-68).

**Conclusion:**

The methanol extract and the ethyl acetate fraction of *P. macrocarpa* fruits exhibited good nutritional values, good antioxidant and cytotoxic activities, and merits further investigation to identify the specific compound(s) responsible for these activities.

## Background

Medicinal plants can be found worldwide. However, they are most abundant in tropical regions. Classifying medicinal plants systematically is important in order to understand and study them extensively and efficiently. The *Phaleria macrocarpa* (Scheff) Boerl plant (local name mahkota dewa) is a medicinal plant that originated from Yogyakarta, Indonesia. The fruits of *P. macrocarpa* have bright red skin, with fruit flesh, shells and seeds located inside the fruit. It has a smooth surface, is round in shape, with sizes ranging from 3-5 cm. It is a drill green color when young and changes to red once ripened. The fruit grows on the trunks and branches of trees with very short stalks. It looks like it is attached to the stem and also has white flesh, and is fibrous and watery.

The major components of *P. marcrocapa* fruits are flavonoids. It also contains alkaloids, saponins, tannins, and terpenoids. The n-hexane extract of *P. macrocarpa* fruits’ flesh contains terpenoids, whereas the ethanol extract of *P. macrocarpa* flesh and seed fruit is composed of alkaloids, flavanoids, and triterpenoids [1]. It has also been shown that the *P. macrocarpa* fruits’ flesh ethyl acetate extract contained flavonoids, triterpenoids and coumarin groups [2].

The fruits of *P. macrocarpa* are most often used in traditional medicine by mixing with other ingredients. It is used to treat a wide variety of diseases such as cancer, diabetes mellitus, allergies, liver and heart diseases, kidney failure, blood diseases, hypertension, stroke, various skin diseases, itching, aches and flu [3,4]. The fruits of *P. macrocarpa* is also likely to possess antimicrobial properties due to the presence of flavonoid compounds [5]. Moreover, a study that examined the effects of mahkota dewa treatment in diabetic animals showed that it was able to decrease the blood glucose level in these animals [6]. Sugiwati reported that extracts of young and ripe fruits of *P. macrocarpa* possessed inhibitory activity against *in vitro* α-glucosidase [7]. In a separate study, it was shown that methanol extracts caused anti-nephropathic action [8]. The ethanol extract of *P. macrocarpa* fruit flesh has been found to be toxic towards the Hela cell line [9].

In the present study, the methanol, hexane, chloroform, ethyl acetate, and water extracts of *P. macrocarpa* fruits were examined for phytochemicals, flavonols, flavonoids, phenol content. Its nutritional values (A.O.A.C method), antioxidant activities (DPPH assay) and cytotoxicity (MTT cell proliferation assay) was also determined.

## Methods

### Plant material

*Phaleria macrocarpa* (Scheff.) Boerl plant meterials were collected from Gowongan Rd., Yogyakarta, Java, Indonesia. A voucher specimen (ID no: KLU 47923) was deposited into a repository at the Rimba Ilmu, Institute of science biological, Faculty of Science, University of Malaya, Malaysia. *P. macrocarpa* fruits were thoroughly washed with fresh water, sliced and dried in an oven at a temperature of no more than 50°C. It was then ground into a fine powder. The dry, finely powdered samples were stored in airtight containers.

### Extraction

The powdered *P. macrocarpa* fruits (1 kg) were soaked in 70% aqueous methanol for three days at room temperature and then filtered to recover the supernatant. The filtrate was then re-filtered and poured into a round-bottomed flask and was evaporated at a low pressure (60 rpm at 37°C) to remove excess methanol. This resulted in a dark-brown coloured, concentrated crude methanol extract.

The methanol extract was then further fractionated with hexane. The hexane-insoluble residue was extracted with chloroform resulting in a chloroform-soluble fraction and a chloroform-insoluble residue. It was then filtered using filter paper and subjected to evaporation under pressure to remove excess solvent. The chloroform-insoluble fraction was then partitioned between ethyl acetate and water. A mixture of ethyl acetate and water (1:1) was prepared and poured into the round-bottomed flask containing the hexane-insoluble extract. It was then filtered using filter paper and subjected to evaporation under pressure to remove excess solvent. The methanol extract and its fractions were removed, placed in a vial, and kept in a refrigerator for further bioactivity assay.

### Phytochemical analysis

The phytochemical constituents of *P. macrocarpa* fruits were determined using chemical methods and by adopting standard protocols as described by Harborne and Evans [10,11].

### Physicochemical determination

Various nutritional values such as moisture, protein, fat, fiber, ash, carbohydrate and sugar contents of the dried, powdered *P. macrocarpa* fruits were determined using the following protocols.

### Moisture determination

The moisture content of the samples was determined by drying 10 g of the sample in an air oven at 105°C until constant weight (16–18 h) was achieved [12]. The crucible containing the dried sample was weighed again and the weight loss was recognized as the moisture content of the dried powdered *P. macrocarpa* fruits. The experiment was repeated three times. The moisture content (%) was calculated by using the following equation:

$$\text{Moisture}\;\text{percentage}\;\left(\%\right)=\frac{W_{2}‒W_{1}}{W_{0}}\times 100$$

Where, W_1_ = weight of sample after drying (g), W_2_ = weight of sample before drying (g),

W_0_ = weight of the sample (g)

### Protein determination

Protein content was determined by using the A.O.A.C method [13]. Briefly, powdered *P. macrocarpa* fruit samples (1 g), 50 mL of distilled water, 5 g of copper sulphate, and 15 mL of concentrated sulphuric acid were added into a Kjeldahl flask. The flask was partially closed by means of a funnel and the contents were digested by heating the flask at an inclined position in the digester. The mixture was heated for approximately 30 minutes until its volume was reduced to 40 mL. Standard sulphuric acid (0.1 M) and a few drops of methyl red indicator were added to the clear solution. The flask was then placed below a condenser and the end of the adapter tube was dipped in the acid. Kjeldahl distillation apparatus was set up and 70 mL of 40% sodium hydroxide was added through the funnel. The funnel was then washed twice with 50 mL of distilled water and distillation was performed for one hour. The distilled ammonia was nitrated with 0.1 M standard solution until the color changed from yellow to colorless. The experiment was repeated three times. The nitrogen content and protein content in the sample was calculated by using the following relation.

$$\text{Percentage}\;\mathrm{of}\;\text{Nitrogen}\;\left(\%\right)=\frac{\left(V_{s}‒V_{b}\right)\times M_{A}\times 0.140\times 100}{\text{Weight}\;\mathrm{of}\;\text{sample}\;\left(W\right)}$$

Proteincontent=percentageofNitrogen×6.25r

Where, V_1_ = volume in cm^3^ of standard acid used in the titration of sample, V_2_ = volume in cm^3^ of standard acid used in the blank titration, M_A_ = molarities of standard acid solution in mol dm^-3^, W = weight of the sample in grams.

### Fat content determination

Fat content was determined using the soxhlet extraction method [13]. Briefly, the powdered *P. macrocarpa* fruits (10 g) were placed in a cotton bag which was then placed in a soxhlet extractor. Petroleum ether was then poured into the soxhlet extractor and the extraction was allowed to proceed for 12 hours. After the extraction was complete, excess petroleum ether was removed by distillation until the volume was reduced to about 15 mL. The residual petroleum ether removed by drying at 105°C in an oven until constant weight was obtained. The experiment was repeated three times. The fat content was then calculated by using the following equation:

$$\%\text{of Fat content}=\frac{\text{Weight}\;\mathrm{of}\;\text{fat}\;\text{obtain}\;\text{from}\;\text{sample}\times 100\;}{\text{Weight}\;\mathrm{of}\;\text{sample}}$$

### Crude fiber determination

Crude fiber content was determined according to the A.O.A.C method [13]. Briefly, dried powdered samples were accurately weighed and placed into a conical flask. Hot sulphuric acid (200 mL, 1.25%) was added and the mixture was boiled for half an hour. Water was added to maintain a constant level. The mixture was then filtered and washed with distilled water. The residue was transferred into a flask with 200 mL of sodium hydroxide solution (1.25%). It was then gently boiled for half an hour. Water was continuously added into the flask to maintain a constant level. The residue was then filtered and washed with boiling water until neutral pH was achieved. It was then washed with alcohol and ether and then dried in an oven at 100°C until constant weight was achieved. The fiber content was calculated by using the following equation:

$$\text{Percentage}\;\mathrm{of}\;\text{Crude}\;\text{fiber}\;\left(\%\right)=\frac{\text{Loss}\;\mathrm{in}\;\text{weight}}{\text{Weight}\;\mathrm{of}\;\text{sample}}\times 100$$

### Ash determination

Ash determination was performed as described by the A.O.A.C method [13]. Briefly, five grams of the dried, powdered sample was weighed in a crucible and then placed in a furnace at 500°C until the substance turned into ash. The crucible was then cooled in a desiccator and weighed. The procedure was repeated until a constant weight was obtained and the percentage of the total ash was calculated using the following formula [14]:

$$\text{Percentage}\;\mathrm{of}\;\text{Ash}\;\left(\%\right)=\frac{\text{Weight}\;\mathrm{of}\;\text{Ash}}{\text{Weight}\;\mathrm{of}\;\text{sample}}\times 100$$

### Carbohydrate determination

Carbohydrate content can be calculated by multiplying reducing sugar content by a factor of 0.9. The reducing sugar content was determined using the Fehling’s reducing method of Lane and Eynon [15]. Weighed samples were placed into 250 mL round-bottomed flasks. Sulphuric acid was then added (20 mL, 0.5 M). Reflux was then performed in a sand bath for 2.5 hours. The flask was then cooled and its content filtered with filter paper. The resulting residue was washed with warm distilled water (a total of 100 mL). A total of 10 mL of the solution was then neutralized with sodium carbonate. The mixture was topped up to 100 mL with distilled water. Fehling’s solution (5 mL) was placed into a flask, followed by 5 mL of distilled water. The solution was boiled for 15 seconds. Several drops of methylene blue indicator was then added and titrated with the sample solution until the color changed from blue to green. The carbohydrate content was then calculated according to the following equation:

$$\text{Percentage}\;\mathrm{of}\;\text{carbohydrate}\;\text{content}\left(\%\right)=\frac{5\times 0.005\times 100\times 100}{V\times 10\times W}\times 0.9\%$$

Where, V = volume of sample solution (titration volume), W = weight of powdered sample

### Reducing sugar determination

Reducing sugar content was determined using the Lane and Eynon titration method [15]. Sample (1 g) was placed in 200 mL of distilled water and shaken for 30 minutes and filtered. Fehling’s A (10 mL) solution and Fehling’s B (10 mL) solution were poured into a flask. The sample filtrate (20 mL) and distilled water (10 mL) were then added. The solution was mixed thoroughly, boiled for 5 minutes, and cooled down to room temperature in an ice bath. This was followed by the addition of 10 mL of 30% potassium iodide solution, 10 mL of 20% sulfuric acid solution and 1 mL of starch indicator solution. Titration was then performed until the blue starch-iodine color disappeared. Reducing sugar content was calculated using the following formula:

$$\begin{array}{ll} \text{Percentage}\;\mathrm{of}\;\text{reducing}\;\text{sugar}\;\left(\%\right)= & \frac{\times 200\;\mathrm{ml}}{\text{Sample}\;\mathrm{wt}\;\left(g\right)\;\times \;20\;\mathrm{ml}\times 1000\mathrm{mg}/g}\\ & \times 100 \end{array}$$

### Phenol determination

The total phenol content of the crude methanol extract and its fractions (hexane, chloroform, ethyl acetate, and water) were determined using the Folin-Ciocalteau method with slight modification [16-19]. Briefly, 200 μl (20mg/mL) of each sample was added to 1 mL of 10% Folin-Ciocalteu reagent and 800 μL of 7.5% Na_2_CO_3_ solution. The mixture was shaken for five minutes and then incubated at 37°C for 15 minutes, followed by incubation in the dark for 1 hr. Absorbance was then measured at 760 nm against distilled water as a blank. Gallic acid was used to construct a standard curve. The amount of total phenol content was calculated as mg/g gallic acid equivalent (GAE). For the gallic acid, the curve of absorbance versus concentration was described by the equation y = 0.00138x - 0.00247 (R^2^ = 0.923), where, y = absorbance and x = concentration [20]”. The Total phenolic content of extracts and fractions of *P. macrocarpa* fruits was calculated using the following formula: Total phenol content = GAEx V/m. Where GAE is the gallic acid equivalence (mg/mL); V is the volume extract (mL) and m is the weight (g) of the pure plant extract.

### Flavonoid determination

The total flavonoid content of the methanol extract of *P. macrocarpa* and its fractions was measured as previously described, with modifications [16,21,22]. Briefly, 1 mL of sample (1mg/mL) was poured into a centrifuge tube. This was followed by the addition of 0.1 mL of 10% Al(NO_3_)_3_ solution, 0.1 mL of 1 M potassium acetate, and 3.8 mL of methanol. The content of the centrifuge tube was then mixed thoroughly with a vortex mixer for two to three minutes and allowed to stand for 10 minutes at room temperature. Absorbance was then measured at 410 nm. Quercetin was used as a standard compound for the quantification of total flavonoids. Total flavonoid content was calculated as quercetin equivalent (mg/g) using the equation obtained from the curve Y = 0.0869x + 0.15, R^2^ = 0.9684, where X is the absorbance and Y is the quercetin equivalent. The amount of flavonoids in the sample in quercetin equivalents was calculated using the formula: X = (A × m_0_ x 10)/(A_0_ x m).Where: X = flavonoid content, mg quercetin/g; A = the absorbance of sample; A0 = the absorbance of standard quercetin.

### Flavonol determination

The method of Kumaran and Mbaebie [23,24] with slight modification was used to measure the total flavonol content. Briefly, 1 mL of extract or fractions (1 mg/mL) was added to a centrifuge tube with 2 mL of AlCl_3_ prepared in ethanol and 3 mL of 50 g/L sodium acetate solution. This was then mixed thoroughly with a vortex mixer and incubated for 1 hour. Absorbance was then measured at 440 nm with a spectrophotometer. Total flavonol content was calculated as quercetin equivalent (mg/g) using the following equation based on the calibration curve Y = 0.0297x + 0.1288, R^2^ = 0.9729, where X is the absorbance and Y is the quercetin equivalent. X = (A × m_0_ × 10)/(A_0_ × m).Where: X = flavonoids content, mg quercetin/g; A = the absorbance of sample; A0 = the absorbance of standard quercetin.

### Antioxidant determination of DPPH assay

DPPH (1, 1 Diphenyl -1- picrylhydrazyl) has a free radical that tends to capture hydrogen from antioxidants. Due to its free radical, the methanolic DPPH is violet and absorbs at 517 nm. Antioxidant activity screening of *P. macrocarpa* fruit extract and fractions was carried out by determining the DPPH free radical scavenging property using the UV spectrophotometric method [21,25]. Briefly, 50 μL of test solutions (different concentrations from dry extracts) were dissolved in water and added to 1.95 mL of DPPH in methanol. The mixtures were vortex-mixed and kept at room temperature, in the dark, for 30 minutes. The increase in absorbance was recorded at 517 nm. Methanol was used as a blank, methanol and DPPH solution was used as a negative control (A0), and gallic acid, a standard phenolic compound, was used as a positive control. The antioxidant activity was represented as IC_50._ The antioxidant activity was determined as the final concentration of the tested sample required for the prevention of the generation of DPPH radical by 50% [26]. The DPPH radical concentration was calculated using the following equation:

$$\text{Scavenging}\;\text{effect}\left(\%\right)=\frac{\left(A0-A1\right)\times 100}{A0}$$

Where, A0 was the absorbance of the control reaction and A1 was the absorbance in the presence of the tested extracts. The IC_50_ (concentration providing 50% inhibition) was calculated graphically using a calibration curve in the linear range by plotting the extract concentration versus the corresponding scavenging effect [26].

### *In Vitro* cytotoxicity screening

#### ***Cancer cell lines and culture medium***

In this study, Ca Ski (human cervical carcinoma cell line), MCF-7 (human hormone-dependent breast carcinoma cell line), HT-29 (human colon carcinoma cell line), SKOV-3 (human ovarian carcinoma cell line), and MDA-MB231 (human hormone dependent breast carcinoma cell line) were obtained from the American Tissue Culture Collection (ATCC, U.S.A). The cells were cultured and routinely maintained in RPMI 1640 medium for the CasKi, MCF-7 and HT-29 cells lines, and in Dulbecco’s Modified Eagle’s medium (DMEM, Sigma) for the SKOV-3 and MDA-MB231 cell line. They were supplemented with 10% fetal bovine serum, 100 μg/mL penicillin or streptomycin (P/S), and 50 μg/mL of kanamycin/amphotericin B. Cell cultures were incubated at 37°C in a humidified atmosphere containing 5% CO_2_. The culture was sub-cultured every two to three days and routinely checked under an inverted microscope (Leica, Germany) for any contamination.

#### ***MTT cell proliferation assay***

By using MTT (3, 4, 5-dimethylthiazol-2-yl)-2-5-diphenyltetrazolium bromide) assay, cell viability was determined. This assay was based on the reduction of MTT by the mitochondrial dehydrogenase of intact cells to a purple formazan product as described by the methods of Denizot and Mosmann [27,28]. Briefly, cells from a confluent tissue culture flask were spun at 1,000 rpm for 5 minutes and re-suspended with 1.0 mL of growth medium. The density of the viable cells was then determined using 0.4% of trypan blue exclusion dye in a haemocytometer with a microscope. The cells were then seeded into the wells of a microtiter plate and incubated in a CO_2_ incubator at 37°C for 24 hours in order to allow the cells to adhere and achieve 70 - 80% confluence. After 24 hours, the media for each extract (different concentrations - 1, 10, 25, 50 and 100 μg/mL) were removed and 200 mL of 10% media were added into the respective wells containing the cells. The assay was carried out in triplicate. Wells with untreated cells were used as the negative control. After incubation, MTT (10 mL) was added to each well [29]. The amount of formazan produced was determined by measuring absorbance at 540 nm using an ELISA micro plate reader.

The percentage of inhibition (%) was calculated according to the following formula:

$$\text{Percentage}\;\mathrm{of}\;\text{inhibition}\;\left(\%\right)=\frac{\mathrm{OD}\;\text{control}-\mathrm{OD}\;\text{sample}\times 100\%}{\mathrm{OD}\;\text{control}}$$

Cytotoxicity was expressed as an IC_50_ value. The IC_50_ value is the concentration of test compounds that causes 50% inhibition or cell death. The averaged values from three experiments were considered and the percentages of inhibition versus concentration of test compounds were plotted. The extract that gave an IC_50_ value of 30 μg/mL or less was considered active [27].

#### ***Statistical analysis***

All data was subjected to statistical analysis by analysis of variance (ANOVA). Data from the three different time points, five cell lines and five different concentrations were analyzed in a combined analysis. For the determination of which concentration was most effective and which cell lines were most affected, a mean comparison was performed in DMRT Duncan (Duncan’s multiple range test). In addition, advanced SAS 9.2 and Minitab 16.2 software system were also used for analyzing data.

## Results

### Extraction

**Figure 1:**
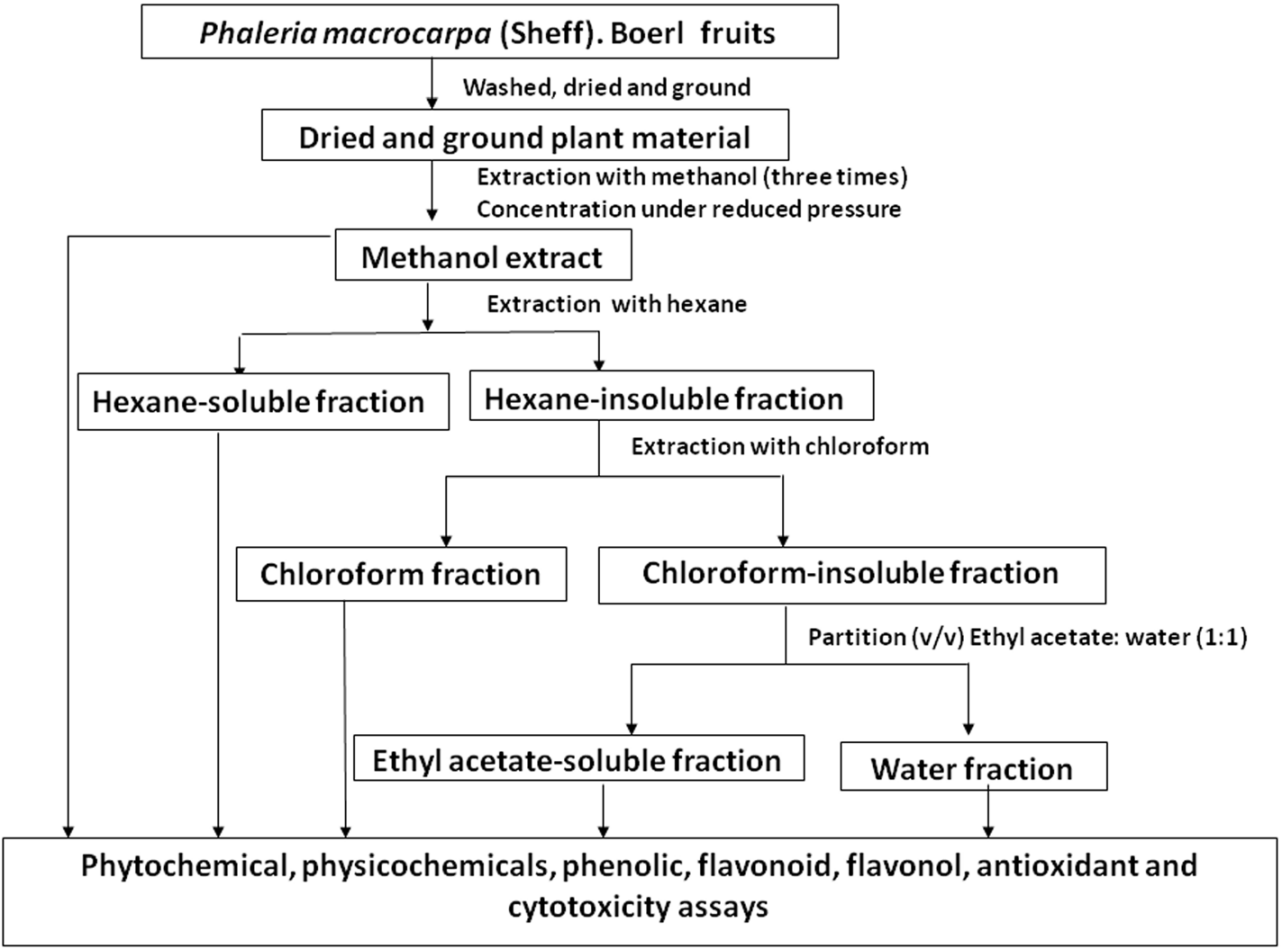
**Summary of extraction and fractionation procedures of *Phaleria macrocarpa *** **(Scheff.) Boerl fruits.**

*P. macrocarpa* fruits were collected in Yogyakarta, Indonesia in 2009. From the air-dried and finely milled samples (1000 g of fruits) hexane, chloroform, ethyl acetate and water extraction were performed sequentially. The extracts were concentrated using a rotary evaporator (Buchi, USA) under reduced pressure at 35°C. Dried extracts were stored at -4°C. A diagrammatic representation of the extraction and fractionation carried out on *Phaleria macrocarpa* (Scheff.) Boerl fruits are shown in Figure 1.

The percentage of crude methanol extract yield was derived from the weight of dried and ground plant material. The hexane extract provided the lowest yield (0.20%) for followed by the chloroform extract (0.43%) as shown in Table 1.

**Table 1:** **Yield of methanol extract and its fractions (hexane, chloroform, ethyl acetate and water) from *P. macrocarpa *** **fruits**

**Extract and fractions**	**Yield (g)**	**Percentage of yield**
Methanolic extract	47.2^a^	4.7
Hexane fraction	2.1^c^	0.20
Chloroform fraction	3.1^c^	0.31
Ethyl acetate fraction	12.2^b^	1.22
Water fraction	Freez dry	

The means of the yield with same small letters were not significantly different as per Duncan’s multi-range test at P < 0.05.

### Quantitative physicochemical screening of *P. macrocarpa* fruits

**Figure 2:**
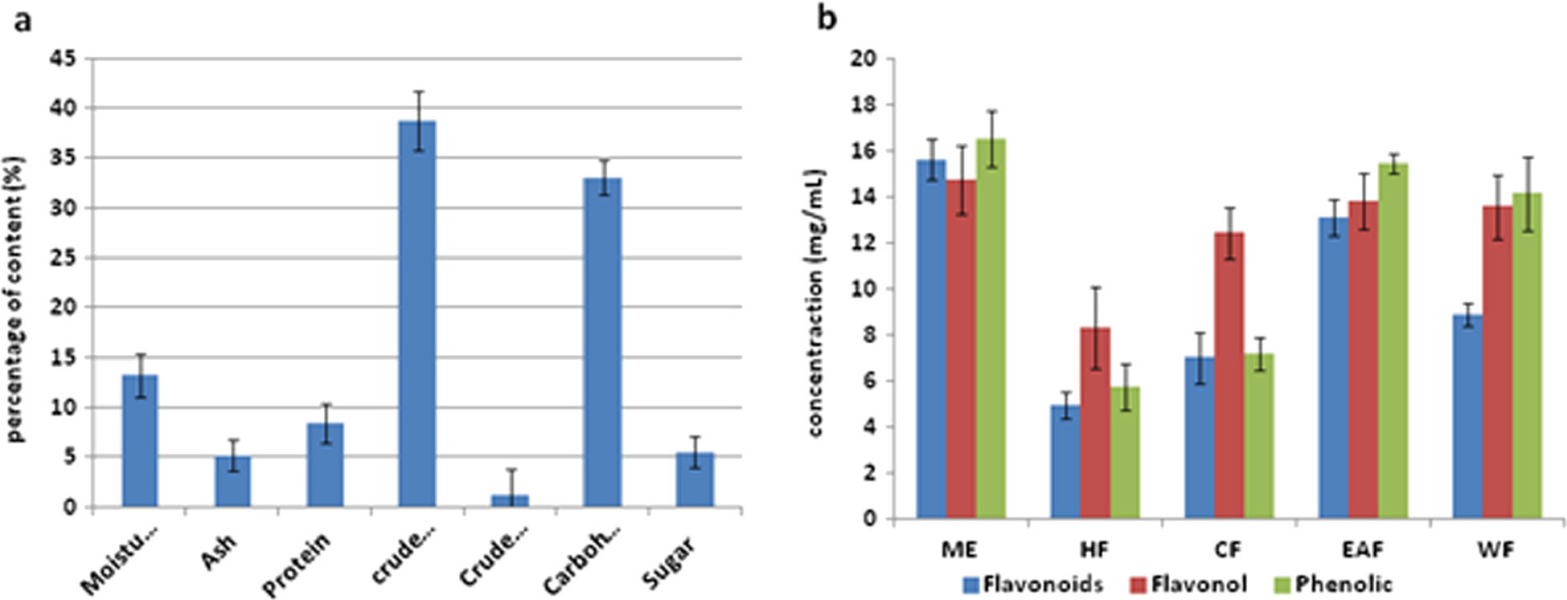
**(a) The percentage of moisture, ash, protein, crude fiber, crude fat, carbohydrate and sugar contents on *P. macrocarpa *fruits; (b) Total amount of concentration of flavonoid, flavonol, phenolic contents on methanol extract and its fractions for *P. macrocarpa *fruits. **(ME: methanol extract; HF: hexane fraction; CF: chloroform fraction; EAF: ethyl acetate fraction; WF: water fraction).

The determination of nutritional values such as moisture, ash, fibre, protein, fat and carbohydrate was carried out using AOAC and, the Lane and Eynon titration methods and the results obtained are shown in Table 2 and Figure 2 (a). Different physicochemical parameters such as moisture content (13.21±2.13%), total ash (5.24±1.56%), proteins (8.51±1.99%), crude fibre (38.77±3.01%), crude fat (1.25±2.65%), carbohydrate (33.02±1.72%) and sugar (5.57±1.49%) were determined.

**Table 2:** **The percentage of moisture, ash, protein, crude fiber, crude fat, carbohydrate and sugar contents and the amount of nutrition values on *P. macrocarpa *** **fruits**

**Test parameter**	**Percentage of contents**
Moisture	13.21±2.13
Ash	5.24±1.56
Protein	8.51±1.99
Crude fiber	38.77±3.01
Crude fat	1.25±2.65
Carbohydrate	33.02±1.72
Sugar	5.57±1.49

### Preliminary phytochemical analysis on *P. macrocarpa* fruits

In order to deterine the types of phyto-organic constituents present in the fruits of *P. macrocarpa*, phytochemical investigation was carried out according to conventional methods, and the results obtained from these experiments are summarized in Table 3.

**Table 3:** **Results of preliminary phytochemical consitutents on *P. macrocarpa *** **fruits**

**No.**	**Tests**	**Reagents**	**Fruit**
1	Alkaloids	Wagner’s Reagent	+
		Mayer’s Reagent	+
		Dragendroff’s Reagent	+
		Sodium picrate solution	+
2	α -amino acids	Ninhydrin Reagent	+
3	Carbohydrates	10% α-napthol and conc: H_2_SO_4_	+ +
4	Cyanogenic glycosides	Sodium picrate solution	-
5	Flavonoids	Mg and conc: HCl	+ + +
6	Glycosides	10% lead acetate	+ + +
7	Organic acids	Bromothymol blue	+
8	Phenolic compounds	1% FeCl_3_	+ + +
9	Reducing sugars	Fehling’s solutions	+
		A and B	
10	Saponin glycosides	Distilled water	+ + +
11	Starch	Iodine solution	+
12	Steroids	Acetic anhydride and conc: H_2_SO_4_	+ +
13	Tannins	1% Gelatin	+ + +
14	Terpenoids	Acetic anhydride and conc: H_2_SO_4_	+ +

+ + + = large amount.

+ = small amount.

+ + = medium amount.

- = absent.

The phytochemical tests showed that secondary metabolites such as flavonoids, glycosides, saponin glycosides, phenolic compounds, steroids, tannins, and terpenoids were present in different extracts of *P. macrocarpa* fruits. Alkaloids, α -amino acids, organic acids, reducing sugars and starch were found to be present in low amounts.

The high amount of phytochemical constituents such as flavonoids (major component), glycosides, saponin glycosides, phenolic compounds, tannins, and terpenoids present in the fruits of *P. macrocarpa* may contribute to the plant’s bioactivities such as anti-oxidant and cytotoxic properties. In addition, there were also medium amounts of carbohydrates, steroids and terpenoids present in the fruits of *P. macrocarpa.*

### Flavonoid, flavonol and phenol determination

In our study, it was found that the methanol extract had the highest flavonoid content which was 15.62±0.9 mg, followed by ethyl acetate, water, chloroform and hexane fractions, which were 13.11±0.8, 8.91±0.5, 7.04±1.1 and 4.98±0.6 mg respectively.

The methanol extract of *P. macrocapa* fruits exhibited the highest amount (14.75±1.5 mg/mL) of total flavonol content, followed by water, ethyl acetate, chloroform and hexane fractions, which were 13.84±1.2, 13.6±1.6, 12.46±1.1 and 8.32±1.8 mg/mL.

The total phenolic content was reported as gallic acid equivalents. The methanol extract of *P. macrocarpa* fruits had the highest total phenol content (16.54±1.5 mg/mL), followed by the ethyl acetate fraction (15.46±0.4 mg/mL) and water fraction (14.15±1.9 mg/mL). The chloroform (7.20±0.7 mg/mL) and hexane fractions (5.75±1.20 mg/mL) had a lower amount of total phenol content.

This data concerning the total amount of flavonoid, flavonol and phenolic content of the methanol extract and its fractions (hexane, chloroform, ethyl acetate and water) of *P. macrocarpa* fruits are shown in Table 4 and Figure 2 (b).

**Table 4:** **Total flavonoid, total flavonol, total phenolic contents and result of DPPH free radical scavenging property of *P. macrocarpa *** **fruits**

**Test parameter**	**ME(mg/ml)**	**HF(mg/ml)**	**CF(mg/ml)**	**EAF(mg/ml)**	**WF(mg/ml)**
Flavonoid	15.62^a^±0.9	4.98^bc^±0.6	7.04^b^±1.1	13.11^a^±0.8	8.91^b^±0.5
Flavonol	14.75^a^±1.5	8.32^c^±1.8	12.46^b^±1.1	13.84^a^±1.2	13.6^a^±1.6
Phenolic	16.54^a^±1.5	5.75^c^±1.2	7.20^c^±0.7	15.46^a^±0.4	14.15^ab^±1.9
DPPH	9.12^b^±0.03	13.01^a^±0.2	13.8^a^±0.03	8.15^b^±0.02	14.1^a^±0.03

(ME: Methanol Extract; HF: Hexane Fraction; CF: Chloroform Fraction; EAF: Ethyl Acetate Fractions; WF: Water Fraction); The means of the each parameters extracts with same small letters were not significantly different as per Duncan’s multi-range test at P < 0.05.

### DPPH antioxidant assay

**Figure 3:**
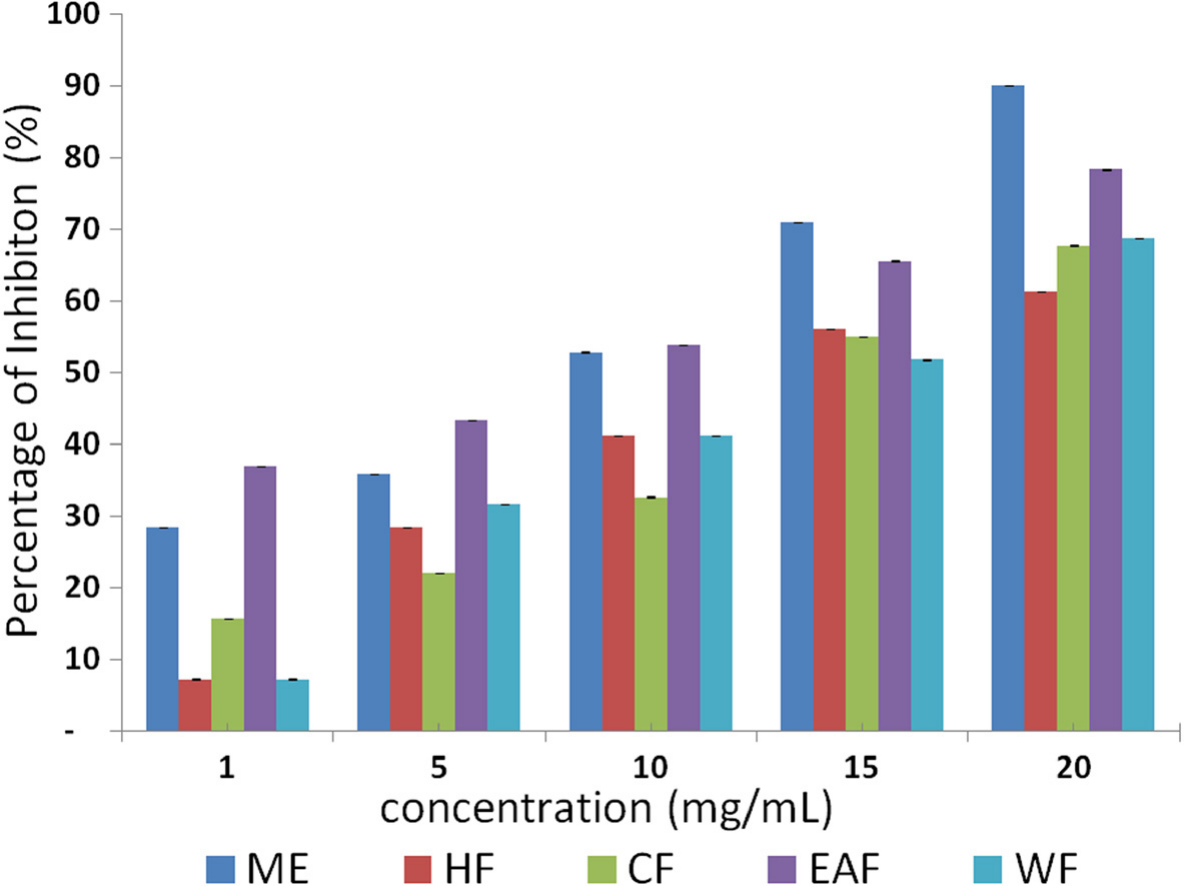
**Percentage of inhibition of DPPH free radical scavenging by the methanol extract and its fractions for *P. macrocarpa *fruits. **(ME: methanol extract; HF: hexane fraction; CF: chloroform fraction; EAF: ethyl acetate fraction; WF: water fraction).

The ethyl acetate fraction of *P. macrocarpa* exhibited the highest free radical scavenging activity followed by the methanol extract, hexane fraction, chloroform fraction and water fraction. A lower IC_50_ value corresponds to higher scavenging activity. Scavenging activity was expressed as a percentage of inhibition of DPPH free radicals (Table 4 and Figure 3). The antioxidant content was reported as gallic acid equivalents and also IC_50_ value of the standard gallic acid was 10.8±0.02 ug/mL comparable to IC_50_ values of all extracts. The results of the DPPH assay also showed that the ethyl acetate fraction with an IC_50_ value of 8.15±0.02 ug/mL and methanol extract with an IC_50_ value of 9.12±0.03 ug/mL of *P. macrocarpa* fruit had a stronger scavenging activity than that of the hexane (13.01±0.2), chloroform (13.8±0.03) and water (14.1±0.03) mg/mL fractions. It was quite interesting to note that the ethyl acetate fraction and methanol extract recorded high inhibitory activities at all the concentrations tested in an increasing order.

The results of analysis of variance (ANOVA) in five different solutions of flavonoid, flavonol and phenolic contents are shown in Tables 5. The effect(s) of the solutions for different contents at 1% level showed significant difference.

**Table 5:** ANOVA of different contents at various solutions

**S.O.V**	**df**	**Phenolic**	**Flavonol**	**Flavonoid**	**DPPH**
Solution	4	0.0141**	0.0168**	0.4322**	0.00032**
Replication	2	1.3E-06	1.70E-07	1.00E-05	2E-07
Error	8	3.5E-06	2.20E-07	1.00E-06	3.00E-06
CV%		16	13	19	12

**: Correlation coefficients between conditions were significant at 1% probability level.

### Cytotoxicity screening on MTT cell proliferation assay

The methanol extract and its fractions (hexane, chloroform, ethyl acetate, and water) of *P. macrocarpa* fruits were investigated for their cytotoxic effects in Ca Ski, MCF-7, HT-29, SKOV-3, MDA-MB231 and normal fibroblast lung cells (MRC-5) and normal liver cells (WRL-68) using MTT cell proliferation assay at 24 hours, 48 hours and 72 hours respectively.

The methanol extract of *P. macrocarpa* fruits showed good cytotoxicity with an IC_50_ value of 20.3±3.71 μg/mL at 24 hrs against a breast cancer cell line (MDA-MB231). The IC_50_ values for the methanol extract measured at 48 hrs and 72 hrs of treatment in MDA-MB231 were 34.6±1.83 and 95±2.11 μg/mL. The methanol extract showed the highest inhibitory effect against ovarian cancer cells (SKOV-3) at 72 hrs (IC_50_ value 7.75±2.56 μg/mL). However, at 24 hrs and 48 hrs of exposure to the methanol extract, IC_50_ values were less impressive (80.0±2.44 and 68.1±1.81 μg/mL respectively). The methanol extract displayed moderate cytotoxic activity in HT-29 cells with an IC_50_ value of 38.5±1.82 μg/mL at 72 hrs and very low cytotoxic effect with IC_50_ values of 83.5±2.52 and 68.1±1.81 μg/mL at 24 hrs and 48 hrs respectively. However, the methanol extract did not exhibit any cytotoxic effect against Ca Ski, and MCF-7 cells with IC_50_ >100 μg/mL. In addition, the extract also did not show any cytotoxic effect on normal MRC-5 cells and WRL-68 cells with IC_50_ >100 μg/mL. Therefore, it can be concluded that the methanol extract exhibited high cytotoxicity in SKOV-3 at 72 hrs and MDA-MB231 at 24 hrs, moderate cytotoxicity at 48 hrs in MDA-MB-231 and HT-29 at 72 hrs, very low cytotoxicity in HT-29 at 24 hrs and 48 hrs and SKOV-3 at 24 hrs and 48 hrs, and no cyctotoxicity in MCF-7, Ca Ski and MRC-5 cells.

The hexane fraction of *P. macrocarpa* fruits showed good cytotoxic effect with an IC_50_ value of 10.15±2.71 μg/mL at 24 hrs in SKOV-3, 5.8±2.15 and 4.6±1.32 μg/mL at 24 hrs and 48 hrs in MDA-MB 231. The IC_50_ value for the treatment of MDA-MB 231 with the hexane fraction was 34.3±2.19 μg/mL at 72 hrs and the IC_50_ value of treatment in SKOV-3 were 69.0±2.56, 72.5±3.13 μg/mL at 24 hrs and 48 hrs. Additionally, this hexane fraction displayed moderate cytotoxic effect on HT-29 cells with an IC_50_ value of 81.5±3.0, 60.0±2.33 and 68.1±3.75 μg/mL at 24 hrs, 48 hrs and 72 hours. Furthermore, there was no cytotoxic effect in Ca Ski or MCF-7 cells except for an IC_50_ value of 44.1±2.38 μg/mL at 48 hrs in MCF-7. The hexane fraction displayed high cytotoxic effect in SKOV-3 at 24 hrs and MDA-MB231 at 24 hrs, moderate cytotoxic effect at 48 hrs in MDA-MB231 at 48 hrs and HT-29 at 72 hrs, very low cytotoxic effect in HT-29 at 24 hrs and 48 hrs, SKOV-3 at 24 hrs and 48 hrs, and no cyctotoxic effect in MCF-7, Ca Ski, MRC-5 and WRL-68. The hexane fraction also exhibited high cytotoxic effect in SKOV-3 at 72 hrs and MDA-MB231 at 24 hrs and 48 hrs, moderate cytotoxic effect in MDA-MB231 at 48 hrs and MCF-7 at 48 hrs, low cytotoxic effect in SKOV-3 at 48 hrs and 72 hrs, HT-29 at 24 hrs, 48 hrs and 72 hrs, and no cytotoxic effect in Ca Ski at 24 hrs, 48 hrs and 72 hrs, as well as in MCF-7 at 24 hrs and 72 hrs.

The chloroform fraction of *P. macrocarpa* fruits exhibited the highest cytotoxic effect with an IC_50_ value of 14.6±1.45, 7.8±1.57 and 15.3±1.72 μg/mL after 24 hrs, 48 hrs and 72 hrs of incubation in MDA-MB 231. The chloroform fraction showed moderate cytotoxic effect with IC_50_ values of 41.2±3.66, 44.0±3.76, 37.0±2.65 μg/mL at 24 hrs, 48 hrs and 72 hrs against HT-29 cells. The chloroform fraction also exhibited moderate cytotoxic effect in SKOV-3 with IC_50_ values of 35.0±1.11, 37.5±1.62, 37.0±2.65 μg/mL at 24 hrs, 48 hrs and 72 hrs. Low cytotoxic activity was observed in SKOV-3 with an IC_50_ value of 80.0±4.56 at 72 hrs. In MCF-7 cells, this choloroform fraction displayed cytotoxic effect with an IC_50_ value of 36.3±2.76 μg/mL at 24 hrs but low cytotoxic effect with an IC_50_ value of 85.5±1.76 μg/mL at 48 hrs with no cytotoxic effect at 72 hrs (IC_50_ >100μg/mL). In contrast, the chloroform fraction did not display any cytotoxic effect against Ca Ski cells and normal MRC-5 and WRL-68 cells (IC_50_ >100 μg/mL). In conclusion, the chloroform fraction exhibited highest cytotoxic effect in MDA-MB 231 cells at 24 hrs, 48 hrs and 72 hrs, moderate cytotoxic effect in MCF-7 cells at 24 hrs, HT-29 cells for 24 hrs, 48 hrs and 72 hrs, and SKOV-3 cells at 24 hrs and 48 hrs, low cytotoxic effect in MCF-7 at 48 hrs and SKOV-3 cells at 72 hrs, and no cytotoxic effect in Ca Ski at 24 hrs, 48 hrs and 72 hrs, and also MCF-7 at 72 hrs.

**Figure 4:**
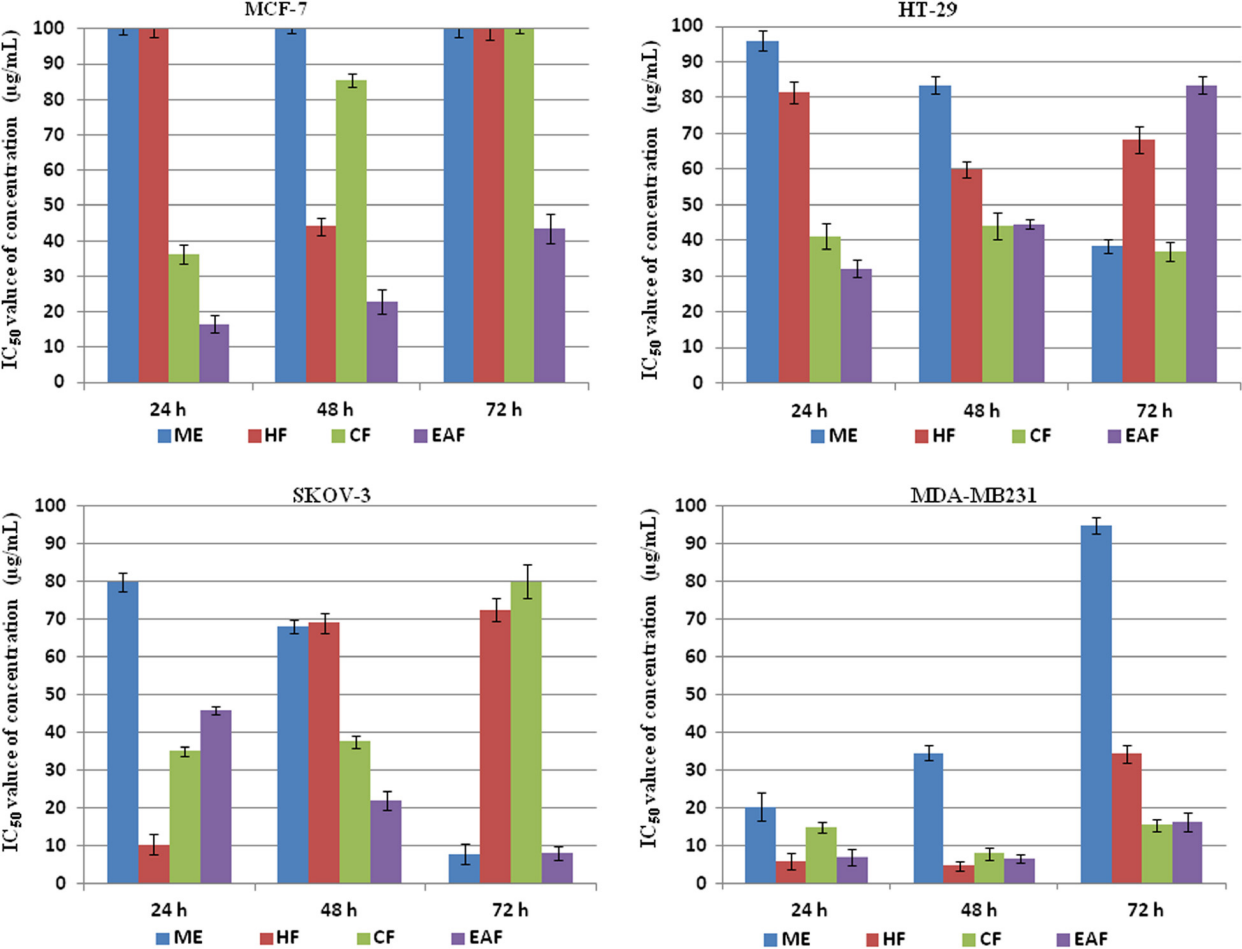
***In vitro*, cytotoxic effects of methanol extract and its fractions of *P. macrocarpa *fruits on MCF-7, HT-29, SKOV-3 and MDA-MB231 cells. **(ME: methanol extract; HF: hexane fraction; CF: chloroform fraction; EAF: ethyl acetate fraction; WF: water fraction).

The ethyl acetate fraction of *P. macrocarpa* fruits showed good cytotoxic effect with IC_50_ values of 21.85±2.58, 8.1±1.81 μg/mL at 48 hrs and 72 hrs in SKOV-3 cells, 6.8±2.08, 6.4±1.09, 16.2±2.4 μg/mL at 24 hrs, 48 hrs and 72 hrs in MDA-MB 231 cells, 16.5±2.45, 23.00±3.44 μg/mL at 24 hrs and 48 hrs in MCF-7 cells. In addition, this ethyl acetate fraction displayed moderate cytotoxic effect in MCF-7 with IC_50_ values of 43.5±4.03 μg/mL at 72 hrs, and 46.0±1.14 μg/mL at 24 hrs in SKOV-3. It exhibited low cytotoxicity in HT-29 cells with an IC_50_ value of 83.5±2.52 μg/mL at 72 hrs. On the other hand, this ethyl acetate fraction was not cytotoxic towards Ca Ski cells, WRL-68 and MRC-5 normal cells. These results are shown in Table 6 and Figure 4.

**Table 6:** ***In vitro*, cytotoxic effects of methanol extract and its fractions of *P. macrocarpa *** **fruits on Ca Ski, MCF-7, HT-29, SKOV3 and MDA-MB231 cancer cells lines**

**Extract**		**ME**	**HF**	**CF**	**EAF**	**WF**
**Cell line**	**hr**					
	24	≥100^a^	≥100^a^	≥100^a^	≥100^a^	≥100^a^
**Ca Ski**	48	≥100^a^	≥100^a^	≥100^a^	≥100^a^	≥100^a^
	72	≥100^a^	≥100^a^	≥100^a^	≥100^a^	≥100^a^
	24	≥100^a^	≥100^a^	36.3^c^±2.76	16.5^c^±2.45	≥100^a^
**MCF-7**	48	≥100^a^	44.1^b^±2.38	85.5^b^±1.76	23.0^b^±3.44	≥100^a^
	72	≥100^a^	≥100^a^	≥100^a^	43.5^a^±4.03	≥100^a^
	24	96^a^±2.92	81.5^a^±3.0	41.2^a^±3.66	32.1^bc^±2.32	≥100^a^
**HT-29**	48	83.5^ab^±2.52	60.0^b^±2.33	44.0^a^±3.76	44.5^b^±1.29	≥100^a^
	72	38.5^b^±1.82	68.1^b^±3.75	37.0^ab^±2.65	83.5^a^±2.52	≥100^a^
	24	80.0^a^±2.44	10.15^b^±2.71	35.0^b^±1.11	46.0^a^±1.14	≥100^a^
**SKOV-3**	48	68.1^ab^±1.81	69.0^a^±2.56	37.5^b^±1.62	21.85^b^±2.58	≥100^a^
	72	7.75^c^±2.56	72.5^a^±3.13	80.0^a^±4.56	8.1^c^±1.81	≥100^a^
	24	20.3^b^±3.71	5.8^b^±2.15	14.6^a^±1.45	6.8^b^±2.08	≥100^a^
**MDA-MB231**	48	34.6^b^±1.83	4.6^b^±1.32	7.8^b^±1.57	6.4^b^±1.09	≥100^a^
	72	95^a^±2.11	34.3^a^±2.19	15.3^a^±1.72	16.2^a^±2.4	≥100^a^

(ME: Methanol Extract; HF: Hexane Fraction; CF: Chloroform Fraction; EAF: Ethyl Acetate Fractions; WF: Water Fraction); The means of the extracts with same small letters were not significantly different as per Duncan’s multi-range test at P < 0.05.

Results of combined analysis of five different concentrations, three time points, five cell lines and interaction among them showed that effect of cytotoxic at probability at 1% level were significantly different (Table 7).

**Table 7:** **Summary of combined analysis and the level of significant mean squares for five cell lines, three hours and five solutions populations of *P. macrocarpa *** **fruits**

**S.O.V**	**df**	**Seq SS**	**Adj MS**	**F**
Cell line (Cell)	4	65420.2	16355	**
Hour (Hr)	2	1572.8	786.4	**
Solution	4	55766.5	13941.6	**
Cell*Hr	8	4196.9	524.6	**
Cell*solution	16	30845.3	1927.8	**
Hr*solution	8	4193.3	524.2	**
Cell*Hr*Solution	32	29067	908.3	**
Error	74	8.80E-05	1.20E-06	

**: Correlation coefficients between conditions were significant at 1% probability level.

The water extract showed no cytotoxic effect in all investigated cancer cell lines and normal MRC-5 cells (IC_50_ >100 μg/mL).

## Discussion

The major nutritional components such as moisture, ash, lipids, proteins, fibres, carbohydrates, and total sugar were found to be in the suitable range along with good energy. In our present study, the crude fibre of *P. macrocarpa* fruits were of a higher percentage compared to its ash, carbohydrate, sugar, proteins and fat contents. The quality and nutritional value of the fruits have a direct relationship with their crude protein content and inverse relationship with its crude fibre content. At the beginning of the growing season, *P. macrocarpa* fruits have the least nutritional value and quality. The quality improves upon maturity. The amount of carbohydrates increases with the completion of the fruit growing stages, while the density of fibre is decreased.

The crude fibre in fruits and vegetables usually consists of cellulose, hemicellulose, lignin, β-glucans, gums and pectin [30]. The high crude fibre content suggested the fruits may be a good source for phenolic antioxidants. This was confirmed by our results.

Many flavonoids and terpenoids are potent antioxidants, anti-inflammatory, anti-bacterial, anti-viral and anti-cancer agents [31]. Phenolics have anti-oxidative, anti-diabetic, anti-carcinogenic, anti-microbial, anti-allergic, anti-inflammtory, anti-mutagenic activities [32,33]. Steroids are known to be important for their cardiotonic activities and possess insecticidal and anti-microbial properties while tannins are known to possess general antimicrobial and antioxidant activities [34]. Saponins are used in treatment of hypercholesterolemia, hyperglycemia, as antioxidants, anticancers, anti-fungal, anti-bacterial, anti-inflammatory and in weight loss [32,35]. Based on preliminary phytochemical studies, the results revealed that the chemical components of *P. macrocarpa* fruit also contained large amounts of flavonoids, phenolics, steroids, tannins, terpenoids, glucosides, and saponins. It also contained small amounts of amino acids, carbohydrates, starch, reducing sugars, alkaloids and steroids. The ethyl acetate extract from the *P. macrocarpa* fruit flesh contained flavonoids, triterpenoids and coumarin groups [2]. The essential oil from *P. macrocarpa* fruit flesh are known to contain octadecane, triclosan, octacosane, diethylester and tributyl citrate [36].

Flavonoids and flavonols have an essential role in drugs particularly those involved in the reduction of cholesterol and fat and in the reduction of the risk of coronary heart disease. Rohyami [37] reported that the methanol extract of *P. macrocarpa* dried fruits was high in total flavonoids. In our investigation, the total flavonoid and flavonol contents of *P. macrocarpa* fruits were highest in the methanol extract, followed by ethyl acetate, water, chloroform, and hexane fractions.

According to previous researches, phenolics have many biological activities such as an antioxidant, antimutagenic, anticarcinogenic, anti-inflammatory and antimicrobial activities. Hendra suggested that the antioxidant activity of the fruits of *P. macrocarpa* was due to the presence of phenolic and flavonoid compounds, the majority of which are kaempferol, myricetin, naringin, quercetin and rutin [5]. Our results showed that phenolic compounds were present at the highest percentage in the methanol extract, followed by ethyl acetate, water, chloroform and hexane fractions.

Rudi [5,9] reported that *P. macrocarapa* fruits have good antioxidant activity due to the presence of flavonoid and phenolic contents. The compound 6, 4'-dihydroxy-4-methoxy benzophenone-2-0-α-D-glucoside isolated from the butanol extract of *P. macrocarpa* fruits was shown to possess good antioxidant activity [38]. Our results showed that the ethyl acetate fraction and methanol extract of *P. macrocarpa* fruits possessed the highest radical scavenging activity, followed by hexane, chloroform, and water fractions. This is consistent with the high phenolic content of the ethyl acetate fraction as phenolics are known to have good antioxidant activity.

It has been shown that *P. macrocarpa* fruits displayed cytotoxic effect on HT-29, MCF-7 and Hela cell lines [5]. Faried [39] have isolated gallic acid from the fruits of *P. macrocarpa* and demonstrated that it could cause cancer cell death in various cancer cells, including the human esophageal cancer (TE-2), gastric cancer (MKN-28), colon cancer (HT-29), breast cancer (MCF-7), cervix cancer (Ca Ski), and malignant brain tumor (CGNH-89 and CGNH-PM). In our study, various cell lines were treated with different concentrations of different sample for 24 hrs, 48 hrs and 72 hrs. The methanol extract, hexane, chloroform, ethyl acetate, and water fractions were found to inhibit MCF-7, SKOV-3, HT-29, and MDA-MB 231 cell proliferation in a time- and dose-dependent manner. However, all extracts and fractions did not exert any cytotoxic effect on Ca Ski cervical cancer cell line, normal human fibroblast lung cells (MRC5) or normal human liver cells (WRL 68).

Taken together, our results showed that the fruits of the *P. macrocarpa* plant have a high content of secondary metabolites including flavonoids, flavonols and phenolics, and good nutritional value which contribute to the excellent antioxidant, anti-inflammatory and cytotoxic activities.

## Conclusion

In this study, the antioxidant and cytotoxic activities of *P. macrocarpan* fruits on selected cell lines were investigated. In addition, the total preliminary phytochemical constituents such as flavonoids, flavonols, phenolic contents and nutritional values were determined. The results showed that the fruit has a high nutritional value, with good antioxidant and cytotoxic activities. The methanol extract of the fruit merits further investigation as it exhibited a host of beneficial qualities. It has the potential to yield compounds that may be developed into chemotherapeutic agents.

## Abbreviations

(EAF): Ethyl acetate fraction; (ME): Methanol extract; (HF): Hexane fraction; (CF): Chloroform fraction; (WF): Water fraction; (MTT): 3, 4, 5-dimethylthiazol-2-yl)-2-5-diphenyltetrazolium bromide; (DPPH): 1, 1 Diphenyl -1- picrylhydrazyl; (g): Gram; (%): Percentage.

## Competing interests

The authors declare that they have no competing interests.

## Authors’ contributions

MML was involved in all experiments and SM was involved statistical analysis. All authors read and approved the submitted version of the manuscript.

## Pre-publication history

The pre-publication history for this paper can be accessed here:

http://www.biomedcentral.com/1472-6882/14/152/prepub
